# Sulfobutylation of Beta-Cyclodextrin Enhances the Complex Formation with Mitragynine: An NMR and Chiroptical Study

**DOI:** 10.3390/ijms23073844

**Published:** 2022-03-31

**Authors:** Bianka Várnai, Ferenc Zsila, Zoltán Szakács, Zsófia Garádi, Milo Malanga, Szabolcs Béni

**Affiliations:** 1 Department of Pharmacognosy, Semmelweis University, Üllői út. 26, H-1085 Budapest, Hungary; bnkvarnai@gmail.com (B.V.); garadi.zsofia@pharma.semmelweis-univ.hu (Z.G.); 2 Research Centre for Natural Sciences, Institute of Materials and Environmental Chemistry, H-1117 Budapest, Hungary; zsila.ferenc@ttk.hu; 3 Spectroscopic Research Department, Gedeon Richter Plc., P.O. Box 27, H-1475 Budapest, Hungary; z.szakacs@richter.hu; 4 CycloLab, Cyclodextrin R&D Ltd., Illatos út. 7, H-1097 Budapest, Hungary; milomalanga@gmail.com

**Keywords:** mitragynine, sulfobutylether-β-cyclodextrin, NMR, circular dichroism, complex stability

## Abstract

Mitragynine (MTR), the main indole alkaloid of the well-known plant *kratom* (*Mitragyna speciosa*), is one of the most studied natural products nowadays, due to its remarkable biological effects. It is a partial agonist on the opioid receptors, and as such relieves pain without the well-known side-effects of the opioids applied in the clinical practice. MTR and its derivatives therefore became novel candidates for drug development. The poor aqueous solubility and low bioavailability of drugs are often improved by cyclodextrins (CyDs) as excipients through host-guest type complex formation. Among the wide variety of CyDs, sulfobutylether-beta-cyclodextrin (SBEβCyD) is frequently used and official in the European and U.S. Pharmacopoeia. Herein, the host-guest complexation of MTR with βCyD and SBEβCyD was studied using chiroptical and NMR spectroscopy. It was found by NMR measurements that MTR forms a rather weak (log*β*_11_ = 0.8) 1:1 host-guest complex with βCyD, while the co-existence of the 2MTR∙SBEβCyD and MTR∙SBEβCyD species was deducted from ^1^H NMR titrations in the millimolar MTR concentration range. Sulfobutylation of βCyD significantly enhanced the affinity towards MTR. The structure of the formed inclusion complex was extensively studied by circular dichroism spectroscopy and 2D ROESY NMR. The insertion of the indole moiety was confirmed by both techniques.

## 1. Introduction

Mitragynine (MTR) is the main alkaloid of *Mitragyna speciosa* [1], known as *kratom* [2]. The plant belongs to the Rubiaceae family and is indigenous to Thailand and Malaysia [3]. The leaf of the plant has been used in South Asia for hundreds of years to treat a variety of illnesses. The plant is traditionally consumed as a tea, or the fresh leaves are chewed directly [4] and regularly used for their stimulating and analgesic effect [5]. At low doses, the leaves are applied for their energizing effects to enhance physical tolerance, while at higher doses, opioid-like effects predominate [6]. In addition, *kratom* is used as a substitute for opium or to treat opioid withdrawal symptoms [7]. Alkaloids of *kratom* are partial agonists at the human μ-opioid receptors. However, they differ from classical opioids like morphine and heroin, since the binding of MTR to the μ-opioid receptor induces activation of the G-protein-coupled signaling cascade without the activation of β-arrestin-2. Activation of β-arrestin-2 is associated with the adverse effects of opioid receptor activation i.e., respiratory depression, constipation, and addiction [8,9]. Regarding the consumption of *kratom*, several withdrawal symptoms have been reported, such as muscle and bone pain, insomnia, anxiety, decreased appetite, and severe craving [10,11].

**Figure 1:**
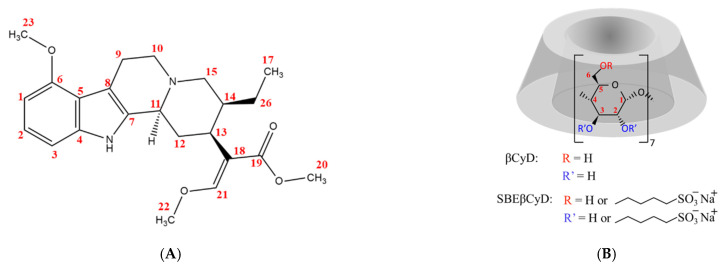
Chemical structure and numbering of mitragynine (**A**) and cyclodextrins used in this study (**B**) (the word “or” was inserted to denote the random substitution in SBEβCyD).

Currently, the most studied alkaloid of the plant is mitragynine, which was isolated and first described by Field et al. in 1921 [12]. The molecule is a tetracyclic indole alkaloid with very poor aqueous solubility (at neutral pH); its structure is shown in Figure 1.

Cyclodextrins (CyDs) are host molecules that are widely used in supramolecular chemistry [13]. They have a truncated cone shape with a hydrophobic cavity and a hydrophilic outer surface that allows them to form inclusion complexes with various guest molecules. This property rendered CyDs to be widely used in pharmaceutical science as excipients [14]. As a result of complex formation, they are frequently utilized to improve the apparent water solubility of highly insoluble compounds, such as the sulfobutylether-β-CyD (SBEβCyD) in the case of the antiviral remdesivir [15]. The internal cavity of CyDs acts as a chiral environment, thus providing the possibility to separate enantiomers by chromatographic or electrophoretic methods [16,17]. Furthermore, CyDs can also behave as catalysts, thereby accelerating chemical reactions [18]. Additionally, complexation with CyDs may result in an increased chemical stability of the guest [19].

The current study aims to understand the inclusion complex formation between MTR and SBEβCyD (see Figure 1 for the structures). Based on preliminary results published earlier for the MTR-βCyD system [20], extensive ^1^H and 2D nuclear magnetic resonance (NMR) as well as circular dichroism (CD) and UV spectroscopic investigations were performed to gain molecular level information (i.e., to determine the stoichiometry of association, the stability constants and the structure of the complex(es) formed) on the MTR-βCyD and MTR-SBEβCyD system. Moreover, detailed interpretation of the CD and the corresponding UV spectroscopic profiles of the free MTR is reported for the first time. The origin and nature of the UV and CD bands, as well as their respective electronic transitions, were discussed and assigned to the chromophoric parts of the molecule.

## 2. Results and Discussion

### 2.1. NMR Spectroscopy

#### 2.1.1. ^1^H NMR Titrations

The complex stoichiometry and stability constants of the CyD-MTR systems were determined by NMR spectroscopy at pH 4.5. The complete ^1^H and ^13^C resonance assignments and registered spectra of uncomplexed MTR are given in the Table S1 and Figure S1 of the Supplementary Material, respectively.

In order to determine the stoichiometry of complexation, an ^1^H NMR titration was performed according to Job’s method [21]. Well-separated NMR signals of MTR were monitored in all experiments. In the case of the native βCyD, Job’s plot curves (Figure S2A) showed a maximum at *x*  =  0.5, suggesting the sole formation of a 1:1 complex. In contrast, several Job’s profiles of MTR with SBEβCyD exhibited maxima at different abscissa values (Figure S2B). These intriguing profiles of chemical shift displacements suggest the coexistence of at least two complex species.

To determine the stability constants of CyD-MTR complexes, a separate ^1^H NMR titration was carried out. During the titration of MTR with βCyD, the chemical shift changes of the following MTR ^1^H resonances were monitored: H1, H2, H3, H11, H12, H12′, H17, and H21, while in the case of the βCyD, H3 was followed. All nine datasets were evaluated simultaneously with nonlinear regression using the OPIUM software [22]. This global evaluation yielded a stability constant of log*K* = 0.8 (0.2), given its estimated standard deviation in parenthesis, indicating a remarkably weak interaction. This weak affinity between MTR and the βCyD is also supported by the fact that despite the use of a tenfold excess of the host during the titration, the inflection point of the titration curve could not be reached (Figure S3). In fact, merely a complexation degree of 2.8% could be achieved. A more precise determination of the formation constant would have required the recording of higher sections of the binding isotherm [23], but titration with a more concentrated stock solution was hindered by the limited water solubility of βCyD.

**Figure 2:**
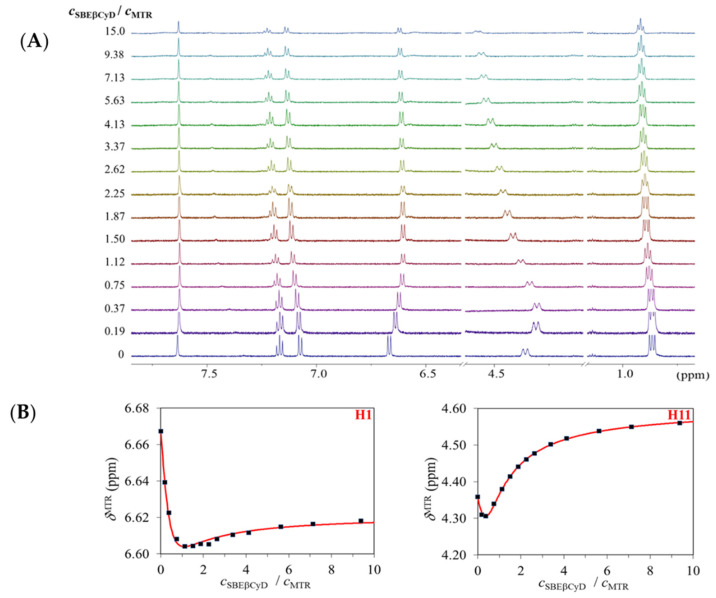
Representative ^1^H NMR multiplet displacements (**A**) of the MTR ^1^H resonances upon titration with SBEβCyD. Subplot (**B**) shows the selected titration profiles of the H1 and H11 MTR resonances. Red curves were fitted by assuming the presence of both the MTR∙SBEβCyD and the 2MTR∙SBEβCyD species.

**Figure 3:**
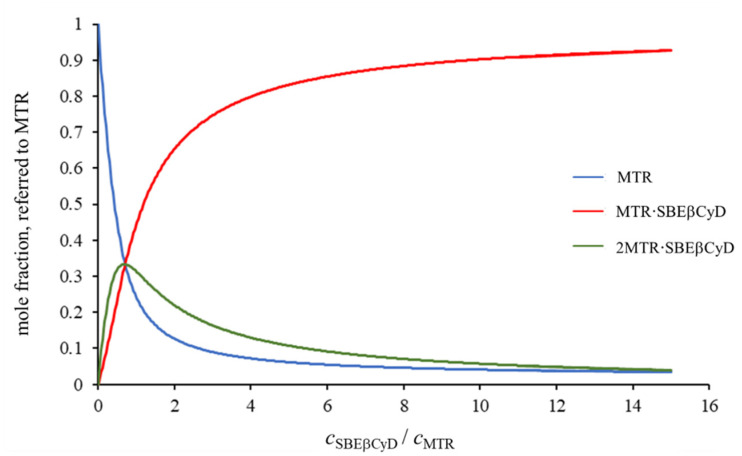
Species distribution plots for the ^1^H NMR titration of MTR with SBEβCyD.

The behavior of the selected MTR signals upon titration with SBEβCyD is depicted in Figure 2A. The non-monotonic displacement of certain multiplets suggests the coexistence of at least two complex species, in accord with the interpretation of the Job’s plots. To obtain reliable association constants, the chemical shifts of H1, H2, H3, H11, H12, and H17 MTR protons were selected to enter the multivariate evaluation. These datasets could not be fitted in a satisfactory manner by assuming either the formation of a single MTR·SBEβCyD or a single 2MTR·SBEβCyD complex. However, employing the two-species {MTR·SBEβCyD, 2MTR·SBEβCyD} model leads to perfect fitting of all the titration datasets, including the non-monotonic ones (see Figure 2B). This global fitting yielded a stability constant of log*β*_21_ = 6.87 (0.10) for the 2MTR·SBEβCyD and log*β*_11_ = 3.68 (0.03) for the MTR·SBEβCyD complex. It is unique, however not unprecedented [24,25], that βCyDs associate with two guest molecules, producing a mixture of 2:1 and 1:1 complexes. The intrinsic chemical shift values of the species are collected in Table S2, and the speciation curves in Figure 3 were constructed for the concentration range explored in the NMR titration. Comparing to the native βCyD, this outstandingly large increase in complex stability could be supported by the possibility of electrostatic interactions between the fully ionized sulfonate groups on the host and the (at pH 4.5) fully protonated cationic guest molecule (MTR has a p*K*_a_ value of 8.1 [26]). The sulfobutylation also provides an enlarged cavity due to the butyl sidechains situated at both the primary and secondary rim of the host, thereby enhancing the encapsulation of the tetracyclic MTR. The flexibility of the sidechains can also contribute to an electrostatically anchored complexation, leading to a much tighter insertion.

#### 2.1.2. Structural Characterization of the Complexes by NMR

**Figure 4:**
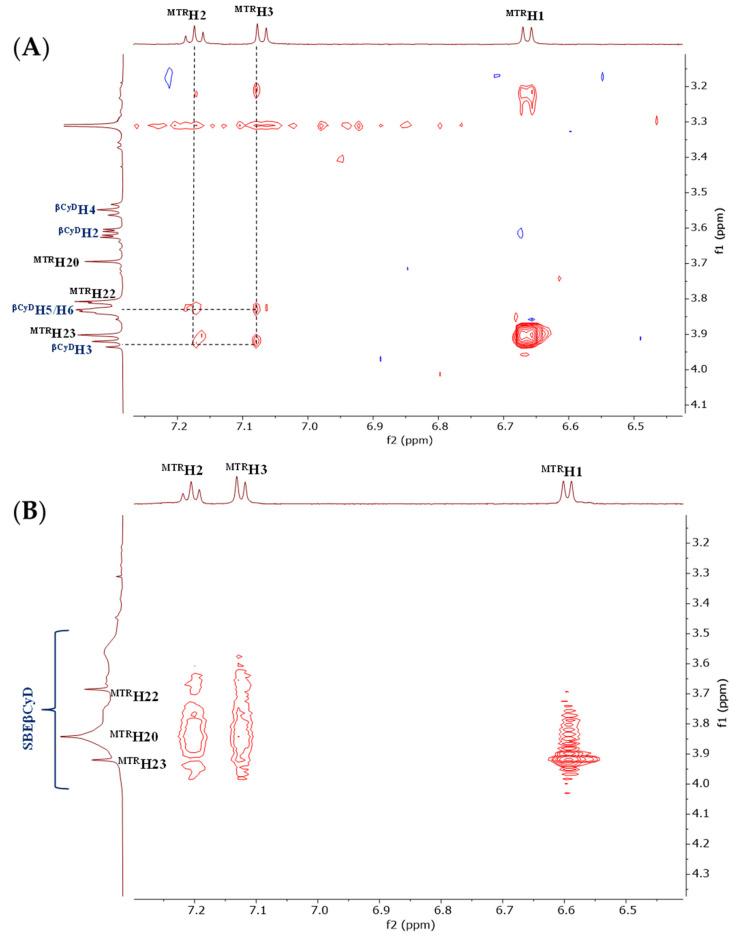
Partial 2D ROESY NMR spectra in the two studied CyD:MTR complexes. Subplot (**A**) (1MTR:2βCyD) molar ratio showing cross-peaks between the aromatic MTR resonances and the inner cavity resonances (H5 and H3) of the βCyD. Subplot (**B**) (1MTR:2SBEβCyD molar ratio) showing diffuse cross-peaks between the aromatic resonances of MTR and the SBEβCyD protons.

To explore the possible structure of MTR-CyD complexes, 2D ROESY spectra were recorded. In the case of βCyD, the host was applied in twofold excess in the NMR sample. Intermolecular NOE cross-peaks could be detected between the MTR’s H1, H2, H3 protons, and the inner cavity resonances of βCyD (H3 and H5), proving that the aromatic moiety is immersed into the host cavity (Figure 4A). The inclusion of MTR occurs from the wider rim of the βCyD.

**Figure 5:**
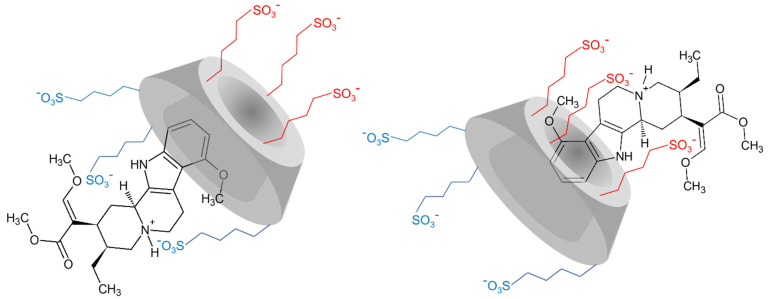
Suggested structure of the MTR∙SBEβCyD inclusion complex based on the registered 2D ROESY NMR spectra and the CD spectroscopic experiments.

Similarly to βCyD, cross-peaks were identified between the aromatic indole ring (H1, H2, H3) and the CyD resonances for the MTR:SBEβCyD sample with 1:2 molar ratio (Figure 4B). However, in this case it is difficult to determine the structure of the complex at atomic level [27], owing to the random distribution of sulfobutyl sidechains at positions O2, O3, and O6 of each glucose unit. Thus, the formation of a 2MTR·SBEβCyD complex could be possible due to the negatively charged sidechains both on the narrower and the wider rim of the CyD. During complexation, the greatest chemical shift displacement values could be observed in the case of the H10 MTR resonance (Δ*δ* > 100 ppb), suggesting that the protonated *N*-heteroatom should be involved in the complexation through electrostatic interaction. The proposed structure of the 1:1 inclusion complex in the MTR·SBEβCyD system is shown in Figure 5. To better understand the possible structure of the 1:1 complex, additional CD spectroscopic measurements were performed.

### 2.2. Circular Dichroism and UV Absorption Spectroscopy

#### 2.2.1. CD and UV Absorption Spectroscopic Properties of Mitragynine

**Figure 6:**
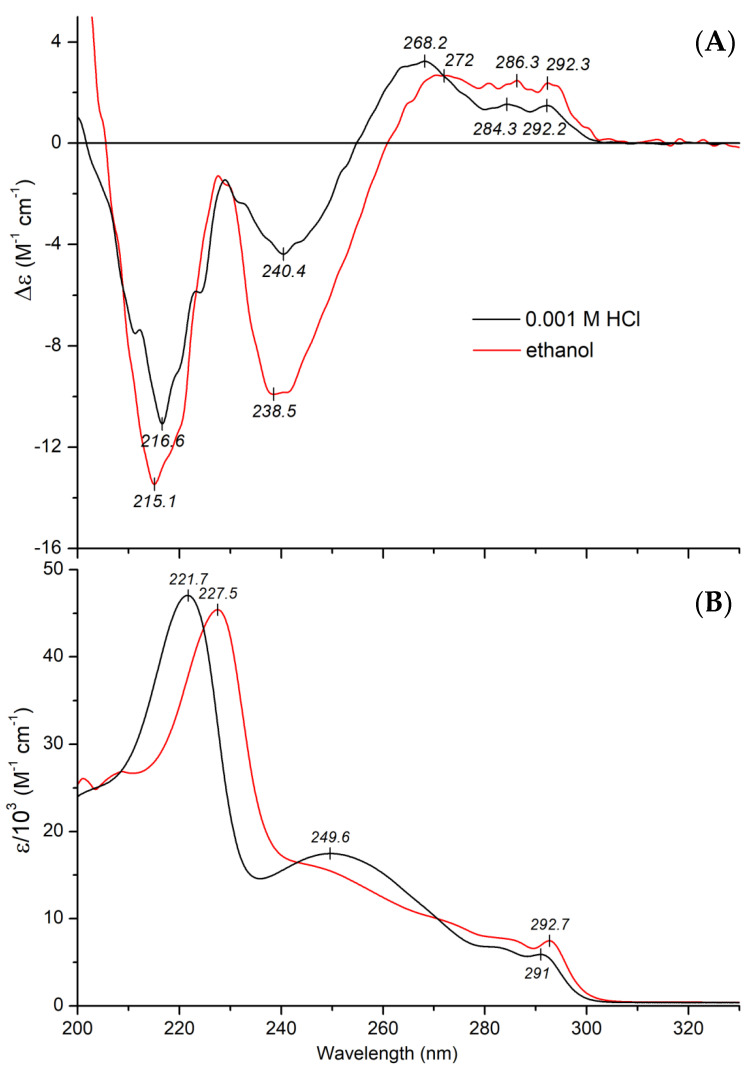
CD (**A**) and UV absorption (**B**) spectrum of 24 μM mitragynine measured in aqueous HCl solution (pH~3) and in ethanol (50 μL stock solution of MTR prepared in 0.001 M HCl was mixed into 1.8 mL EtOH).

Additive-free, acidic aqueous and ethanolic solution of protonated mitragynine displays several Cotton effects (CEs) of various sign allied to the respective absorption bands measured between 200–350 nm (Figure 6A,B).

Based on stereochemical considerations, three different spheres can be defined for chiral aromatic compounds: the chromophore itself forms the first, any ring incorporating the chromophore the second, and groups or rings attached to the second sphere constitutes the third [28]. The term ‘chromophore’ used here includes any double bond and conjugated aromatic or non-aromatic π-systems. The chiral sphere which is nearest to the chromophore chiefly determines the sign and magnitude of the CEs. In the case of MTR, the aromatic π-system of the planar indole ring and the conjugated double bonds of the methoxyacrylate moiety represent the first sphere, whereas the quinolizidine ring system comprises the second and the third spheres. Considering the chemical structure of MTR, the chiral center at C11 being nearest to the indole moiety represents the second sphere. Therefore, its great impact on the indole electronic transitions can be proposed. In line with this, the absolute configuration of C11 determines the CD pattern between 250 and 300 nm: the *S* configuration in MTR correlates with positive CEs but negative signals were obtained for related alkaloids, owing to the *R* configuration at C11 for speciociliatine and mitraciliatine [29,30].

The positive CD feature bearing a vibrational fine structure above 280 nm (Figure 6A) originates from the electric-dipole forbidden ^1^*L_b_* excitation of the indole chromophore [31]. Note that the corresponding π-π* band in the UV spectrum shows similar vibronic peaks with comparable spacing and intensities (Figure 6B). The adjacent, shorter-wavelength positive CE centered around 268 nm (in HCl) can be attributed to the optically active *n*-π* transition of the methyl-3-methoxyacrylate chromophore, in which the C=C–C=O unit is not exactly coplanar but arranged in a transoid fashion [29,32]. This assignment is supported by the 4 nm red shift of the peak observed in ethanol (Figure 6A). The lowering of excitation energy of carbonyl *n*-π* transitions in less polar solvents is a diagnostic feature that enables the identification of corresponding CD signals [33,34]. It is to be noted that the methoxyacrylate moiety has an additional, dipole-allowed π-π* transition, with a λ_max_ value around 230 nm (ε_max_~12,000 M^−1^ cm^−1^) [35,36]. This merges with the adjacent ^1^*L_a_* indole transition [31], making an unusually broad absorption band extending from ~235 to ~275 nm (Figure 6B). Due to this strong spectral overlap, the CEs of these transitions are mixed with each other, producing a net negative CD band with a maximum of around 240 nm (Figure 6A). Presumably, it contains contributions both from the indole (^1^*L_a_*) and the methoxyacrylate π-π* transition. It is also worthy of note that intramolecular exciton coupling may occur between the π-π* dipole moments of the methoxyacrylate and the indole chromophore (^1^*L_a_* and ^1^*B*_b_), giving rise to additional CD activity in the 230–250 nm wavelength range. Due to the asymmetric centers of the quinolizidine ring, these π-systems might be chirally oriented relative to each other, allowing the interaction of their local transition dipoles, which results in CEs in the respective spectral regions [37]. Interestingly, magnitude of the 240 nm CE doubled in ethanol (Figure 6A). Such a large intensification may be attributed to some solvent dependent conformational rearrangement of the quinolizidine ring system, which in turn also affects the indole-methoxyacrylate mutual steric disposition.

The far-UV absorption region of MTR is dominated by the most intense, electrically allowed ^1^*B*_b_ band of the indole ring, which can be decomposed into several Gaussian components (Figure 6B) [31,38]. Allied to this transition, a negative CD band can be seen decorated with vibrational fine structure (Figure 6A). In ethanolic solution, this absorption band exhibits a remarkable bathochromic shift (Figure 6B), but the position of the CE remains invariant (Figure 6A). Therefore, it seems that the π-π* transition responsible for this negative CD signal is buried under the short-wavelength tail of the UV band, and its excitation energy does not show solvent dependence.

#### 2.2.2. CD/UV Spectroscopic Evaluation of Inclusion Complexes of Mitragynine with Sulfobutylether-β-Cyclodextrin (SBEβCyD)

**Figure 7:**
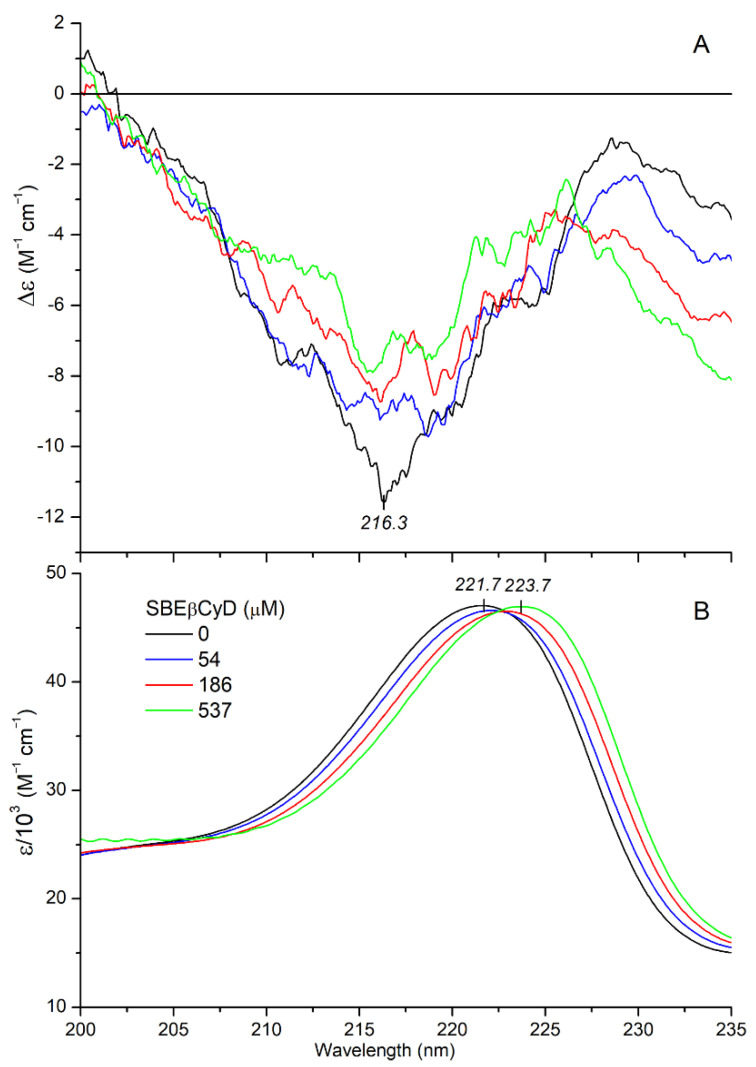
Far-UV CD (**A**) and absorption (**B**) spectrum of 24 μM mitragynine measured in aqueous HCl solution (pH~3) at increasing concentrations of SBEβCyD.

Cyclodextrins are chiral molecular containers as they are composed entirely of α-glucopyranose units in a centrosymmetric arrangement, forming an internal pocket. Therefore, achiral guest molecules often show induced CD signals around their UV/Visible absorption bands upon encapsulation within the CyD cavity [39,40,41]. In addition, the intrinsic CD pattern of chiral compounds may be modified on inclusion due to conformational adaptation and/or interaction of their electronic transition dipoles with those of the σ-bonds of D-glucosyl units [42,43,44]. Accordingly, it is reasonable to think that CyD binding of MTR generates some CD spectral alterations. In order to test this assumption, CD/UV spectroscopic titrations were performed by mixing of SBEβCyD into acidic aqueous solution of the alkaloid. In parallel with the increasing host concentration, λ_max_ value of the ^1^*B*_b_ absorption band exhibited a progressive red shift (Figure 7B).

**Figure 8:**
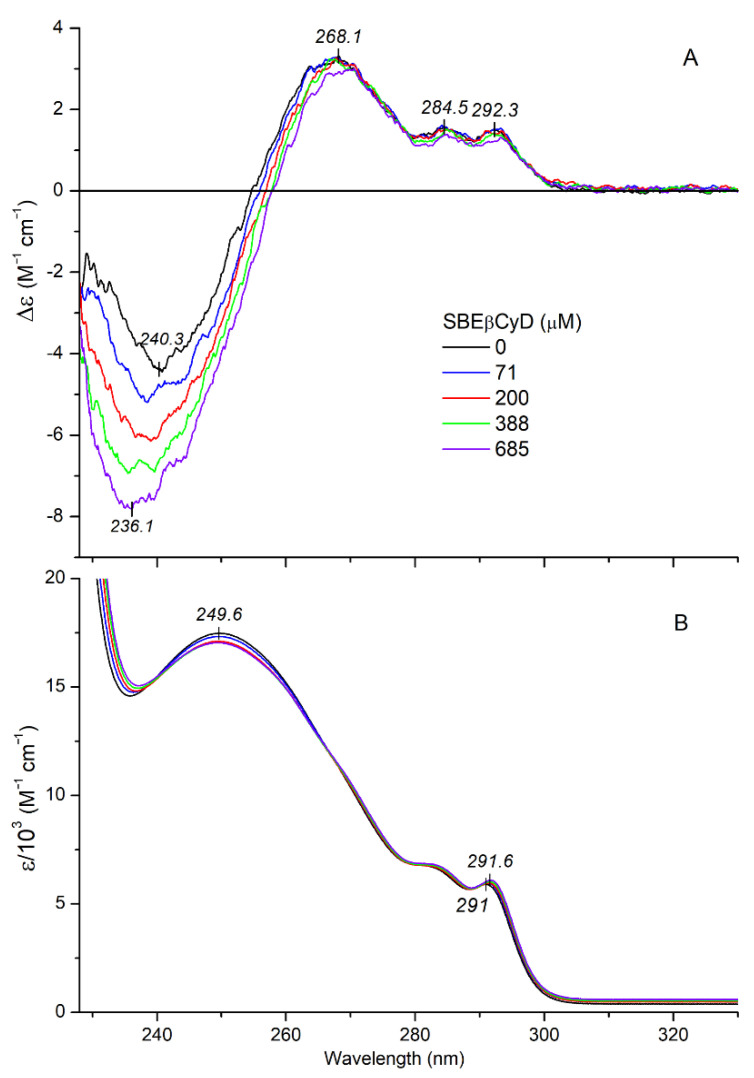
CD (**A**) and absorption (**B**) spectrum of 58 μM mitragynine measured above 228 nm in aqueous HCl solution (pH~3) at increasing concentrations of SBEβCyD.

A similar but somewhat greater shift was observed for free MTR upon changing the polar aqueous environment to the less polar solvent ethanol (Figure 6B). Consequently, the bathochromic shift obtained by addition of SBEβCyD is indicative of the inclusion of the indole moiety into the apolar cavity of the host. Taking into consideration the molecular exciton model [37,39], this situation enables dipole-dipole coupling between the π-π* transition moments of the encapsulated indole ring and the weak electronic moments induced by the oscillating electric field of light in the σ-bonds of the chiral glucose units. According to the empirical sector rule elaborated by Kajtár, the sign of the induced CE is determined by the relative steric orientation of the host-guest dipole moments [39]. A positive CD signal is induced if the transition dipole of the chromophore is oriented parallel to the symmetry axis of the host. Conversely, perpendicular transitions produce negative CEs. However, at an angle of about 30–40° between the direction of the transition moment and the symmetry axis, contributions of the different oscillator pairs cancel each other out and thus no CD activity can be measured. In the case of inclusion complexes of chiral compounds, the CEs induced by this mechanism are superimposed on the intrinsic CD signals of the guest, rendering them stronger or weaker [42,44]. Upon increase in SBEβCyD concentration in the MTR solution, the magnitude of the negative CE at 216 nm reduced by about 30% (Figure 7A). According to this result, the respective transition moment of the indole nucleus is approximately parallel to the C7 symmetry axis of the cyclodextrin, and thereby generates a positive CD contribution [39]. Internal dimensions of SBEβCyD does not allow the entrapment of the entire MTR molecule. As the red shift of the ^1^*B*_b_ band indicated (Figure 7B), the hydrophobic indole ring is absorbed by the apolar cyclodextrin cavity whereas the methoxyacrylate moiety should stick out of it into the bulk solvent. In concert with this proposal, the *n*-π* CE of the methoxyacrylate chromophore does not show any changes compared to the free state, even at the high molar excess of SBEβCyD (Figure 8A cf. Figure 6A). Accordingly, the carbonyl group is not encapsulated into the apolar pocket. However, insertion of the indole part and the protonated nitrogen-sulfate group ionic interaction may affect the structural mobility of the methoxyacrylate moiety, too. Its rotational freedom with respect to the aromatic core of MTR might be reduced upon the complex formation. In this way, the relative contribution of the conformer population responsible for the negative CD motif between 228–255 nm raises, and in turn this enhances the corresponding CD values (Figure 8A). The slight red shift of the highest-wavelength ^1^*L_b_* vibrational peak in the absorption spectrum should also be noted (Figure 8B). Similarly to the shift of the ^1^*B*_b_ band, it refers to the entrapment of the indole ring within the cyclodextrin cavity. The magnitude of the ^1^*L_b_* CEs changed only in a minimal extent. Applying the sector rule mentioned above, this suggests that in relation to the C7 symmetry axis of the CyD, the ^1^*L_b_* transition moment occupies a steric position, which produces negligible induced CD activity.

**Figure 9:**
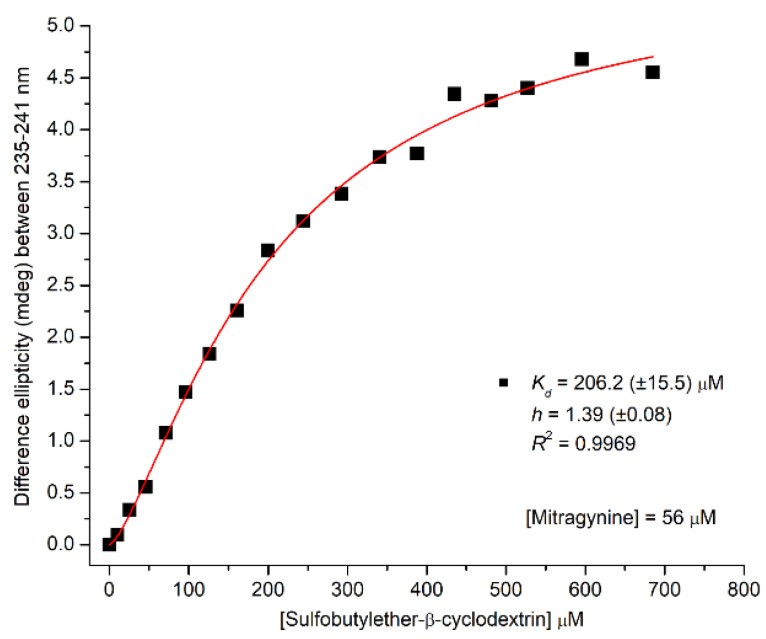
Difference CD values of MTR plotted as the function of SBEβCyD concentrations. Red solid line was obtained by non-linear curve fitting analysis for “one site—total and nonspecific binding” model using the Prism 6 software. Estimated value of the apparent dissociation constant and the Hill coefficient are shown.

The concentration dependent intensification of the methoxyacrylate associated CE enabled to estimate the apparent dissociation constant of the inclusion complex. Non-linear regression analysis was performed on the difference CD maxima of MTR plotted against the rising host concentrations (Figure 9). The calculations yielded a *K*_d_ of 206 μM, which is identical to that obtained from ^1^H NMR experiments (log*β*_11_ = 3.68). The Hill coefficient is larger than 1, suggesting the positive cooperativity of MTR-cyclodextrin binding interaction.

## 3. Materials and Methods

### 3.1. Chemicals and Reagents

MTR was isolated and purified by centrifugal partition chromatography (CPC) from *kratom* extract, and it was a generous gift from RotaChrom Ltd. (Dabas, Hungary). Native βCyD and randomly substituted SBEβCyD (DS~6.5) were the products of CycloLab Ltd. (Budapest, Hungary). D_2_O (99.9 atom% D) was purchased from Merck (Darmstadt, Germany), while acetic acid-d4 was obtained from Cambridge Isotope Laboratories Inc (Tewksbury, MA, USA). Other base chemicals of analytical grade were from commercial suppliers and used without further purification.

### 3.2. NMR Spectroscopic Measurements

NMR spectroscopic measurements were carried out on a 600 MHz Varian DDR NMR spectrometer (Agilent Technologies, Palo Alto, CA, USA), equipped with a 5 mm inverse-detection probehead and a pulsed-field gradient module. Standard pulse sequences and processing routines available in VnmrJ 3.2 C/Chempack 5.1 and MestreNova 14.2.0 were used. All samples were prepared in an acetate buffer (the pH of 20 mM CD_3_COOD solution in D_2_O was adjusted with NaOD to 4.5) and NMR spectra were acquired in standard 5 mm NMR tubes at 298 K. ^1^H chemical shifts were referenced to the methyl singlet (*δ*  =  3.31 ppm) of internal CH_3_OH. The complete ^1^H and ^13^C resonance assignments of mitragynine were established from 1D ^1^H, and ^13^C, 2D ^1^H-^1^H gCOSY, zTOCSY (mixing time of 150 ms), NOESY (mixing time of 300 ms), ^1^H-^13^C gHSQCAD (^1^*J*_CH_ = 140 Hz) and gHMBCAD (^n^*J*_CH_ = 8 Hz) experiments.

#### 3.2.1. ^1^H NMR Titration Experiments

^1^H NMR titration of MTR was carried out according to Job’s method [21] in order to investigate the stoichiometry of MTR complexation with CyDs. Samples were dissolved in 20 mM acetate buffer of pH 4.5 at 298 K. The total molar concentration of MTR and the host component $c_{\mathrm{MTR}}$+ $c_{\mathrm{CyD}}$ was kept constant at 1 mM. The mole fraction of MTR, $x_{\mathrm{MTR}}=c_{\mathrm{MTR}}$/$\left(c_{\mathrm{MTR}}+c_{\mathrm{CyD}}\right)$ was increased in 0.1-unit steps from 0 to 1. ^1^H chemical shifts $\delta_{\mathrm{MTR}\;}^{\mathrm{obs}}$ were recorded for several resonances of MTR, and complexation-induced displacement values Δ$\delta_{\mathrm{MTR}\;}^{\mathrm{obs}}$ = $\left|\delta_{\mathrm{MTR}\;}^{\mathrm{obs}}-\delta^{\mathrm{MTR}}\right|$ were calculated with respect to the $\delta^{\mathrm{MTR}}$ chemical shift of the free guest to construct Job’s plots $\Delta \delta^{\mathrm{MTR}}x_{\mathrm{MTR}}$ vs. $x_{\mathrm{MTR}}$.

To determine the stability constant (an apparent and averaged value in the case of the randomly substituted SBEβCyD), separate NMR titrations were performed under the same experimental conditions, as used for the Job’s plot. For the native βCyD, 500 μL 1 mM MTR was titrated with increasing portions (5–1000 μL) of 10 mM βCyD stock solution, while in the case of SBEβCyD 500 μL 1.05 mM MTR was titrated with 9.88 mM SBEβCyD. Following equilibration, ^1^H NMR spectra were recorded for each titration step at 600 MHz and 298 K. The experimental titration curves for well-resolved resonances of MTR (and for βCyD, that of its H3 proton) were simultaneously evaluated by the OPIUM software [22].

In the case of βCyD, the nonlinear titration fitting was performed by assuming the formation of a single MTR·βCyD (1:1) complex [23]. In contrast, the titration curves with SBEβCyD were fitted according to the principles summarized here by assuming the formation of both MTR·CyD and 2MTR·CyD complexes (the rationale for these species is explained in Results and Discussion). If a complexation occurs with rapid kinetics on the NMR chemical shift timescale, the observed chemical shift $\delta_{\mathrm{MTR},i\;}^{\mathrm{obs}}$ of the *i*th carbon-bound proton in MTR becomes a mole-fraction weighted average [23] of the species-specific values in the uncomplexed MTR ($\delta^{\mathrm{MTR},i}$) and those in the complexes ($\delta^{\mathrm{MTR}\cdot \mathrm{CyD},i}$ and $\delta^{2\mathrm{MTR}\cdot \mathrm{CyD},i}$),

(1) $$\delta_{\mathrm{MTR},i\;}^{\mathrm{obs}}=\frac{\left[\mathrm{MTR}\right]\delta^{\mathrm{MTR},i}+\left[\mathrm{MTR}\cdot \mathrm{CyD}\right]\delta^{\mathrm{MTR}\cdot \mathrm{CyD},i}+2\left[2\mathrm{MTR}\cdot \mathrm{CyD}\right]\delta^{2\mathrm{MTR}\cdot \mathrm{CyD},i}}{c_{\mathrm{MTR}}}$$

where square brackets denote equilibrium concentrations. Analogously defined intrinsic chemical shifts ($\delta^{\mathrm{CyD},j}$, $\delta^{\mathrm{MTR}\cdot \mathrm{CyD},j}$, $\delta^{2\mathrm{MTR}\cdot \mathrm{CyD},j}$) and resonance signal averaging apply for the *j*th carbon-bound proton of the cyclodextrin:

(2) $$\delta_{\mathrm{CyD},j\;}^{\mathrm{obs}}=\frac{\left[\mathrm{CyD}\right]\delta^{\mathrm{CyD},j}+\left[\mathrm{MTR}\cdot \mathrm{CyD}\right]\delta^{\mathrm{MTR}\cdot \mathrm{CyD},j}+\left[2\mathrm{MTR}\cdot \mathrm{CyD}\right]\delta^{2\mathrm{MTR}\cdot \mathrm{CyD},j}}{c_{\mathrm{CyD}}}$$

The mass-balance equations for both constituents read:

(3) $$c_{\mathrm{MTR}}=\left[\mathrm{MTR}\right]+\left[\mathrm{MTR}\cdot \mathrm{CyD}\right]+2\left[2\mathrm{MTR}\cdot \mathrm{CyD}\right]$$

(4) $$c_{\mathrm{CyD}}=\left[\mathrm{CyD}\right]+\left[\mathrm{MTR}\cdot \mathrm{CyD}\right]+\left[2\mathrm{MTR}\cdot \mathrm{CyD}\right]$$

which can be reformulated in terms of the cumulative β association (i.e., binding or formation) constants of the complexes, yielding:

(5) $$c_{\mathrm{MTR}}=\left[\mathrm{MTR}\right]\left(1+\beta_{11}\left[\mathrm{CyD}\right]+2\beta_{21}\left[\mathrm{MTR}\right]\left[\mathrm{CyD}\right]\right)$$

(6) $$c_{\mathrm{CyD}}=\left[\mathrm{CyD}\right](1+\beta_{11}\left[\mathrm{MTR}\right]+\beta_{21}{\left[\mathrm{MTR}\right]}^{2})$$

Based on the known total concentrations $c_{\mathrm{MTR}}$ and $c_{\mathrm{CyD}}$ for each titration step as well as initial guesses of the β stability constants, the OPIUM program [22] solved the nonlinear system of Equations (5) and (6) for the variables [MTR] and [CyD]. These speciation calculations were integrated into a simultaneous least-squares fitting procedure of Equations (1) or (2) to the measured datasets in order to iteratively refine the stability constants. From the resulting complex stability constants, the Microsoft Excel program (and for SBEβCyD, also the Hyss software [45]) was used to generate the fitted NMR titration curves and the species distribution plots.

#### 3.2.2. Structural Characterization of the MTR-CyD Complexes by 2D NMR Measurements

To explore the spatial arrangement of the host-guest complexes, nuclear Overhauser (NOE) effect type experiments [46] were performed on MTR:CyD samples of 1 mM and 2 mM concentration, dissolved in the acetate buffer of pH 4.5. 2D ROESY spectra were acquired, collecting 16 and 32 scans on 1258∙512 data points, applying mixing times of 350 and 400 ms.

### 3.3. Circular Dichroism and UV Absorption Spectroscopic Measurements

CD and UV absorption spectra were acquired in 1 cm path-length quartz cuvette (Hellma, USA) at 25 ± 0.2 °C on a JASCO J-715 spectropolarimeter equipped with a Peltier thermostat. All spectra were recorded in continuous scanning mode at a rate of 50 nm/min, with a step size of 0.1 nm, response time of 2 s, four accumulations and 1 nm bandwidth. UV absorption curves were obtained by conversion of the high-tension (HT) voltage applied to the photomultiplier tube into absorbance units. CD and UV spectra of mitragynine were corrected by the spectral contribution of blank aqueous or ethanol solvent.

CD/UV spectroscopic titration was performed by successive addition of 10 mM SBEβCyD stock solution to the aqueous sample of 58 and 24 µM mitragynine prepared in 0.001 M hydrochloric acid solution.

#### Analysis of CD Titration Data for Estimation the Apparent *K*_d_ Value of Inclusion Complexes

Non-linear curve fitting analysis of the CD titration data was performed by the Graph Pad Prism software (ver. 6.01, San Diego, CA, USA). The following equation was used to fit CD data as a function of the host concentration:

(7) $$y=\frac{B_{\max\;}x^{h}}{K_{d}{}^{h}x^{h}}$$

where *y* is the difference induced CD values of MTR (millidegree) measured at the negative maximum between 235–241 nm, *B*_max_ is the maximum number of binding sites, *h* is the Hill coefficient, *K*_d_ is the equilibrium dissociation constant (μM), and *x* is the SBEβCyD concentration (μM) increased gradually in the sample solution.

## 4. Conclusions

In the present study, interactions of MTR with CyDs were characterized extensively by NMR and CD spectroscopy. ^1^H NMR titration studies revealed a weak (log*β*_11_ = 0.8; MTR٠βCyD) binding between MTR and βCyD, while in the case of SBEβCyD a much stronger interaction was identified (log*β*_11_ = 3.68, log*β*_21_ = 6.87). For the latter case, the co-existence of 1:1 and 2:1 MTR-SBEβCyD complexes was deduced. The CD titration experiments yielded a *K*_d_ of 206 μM, which is in a good agreement with the NMR spectroscopic results. Furthermore, the Hill coefficient (>1) also suggested the prevalence of complexes exceeding the simple 1:1 stoichiometry. Sulfobutylation of βCyD yielded a 750-fold increase in supramolecular stability due to the enlarged cavity, sidechain flexibility, and the possibility of host-guest electrostatic interaction. The structure of the complex was proposed based on CD and NMR data. Both techniques revealed the insertion of the indole moiety into the CyD cavity. Moreover, some NMR and CD spectral signatures were described herein, which are characteristic to the MTR-CyD complex formation. As such, they could be utilized in the future for detection and evaluation of various inclusion complexes of structurally related indole and oxindole *kratom* alkaloids.

## Data Availability

Not applicable.

## Supplementary Materials

The following supporting information can be downloaded at: https://www.mdpi.com/article/10.3390/ijms23073844/s1.
