# Supplementary material for: Sulfobutylation of Beta-Cyclodextrin Enhances the Complex Formation with Mitragynine: An NMR and Chiroptical Study
Source: Int J Mol Sci. 2022 Mar 31;23(7):3844. doi: 10.3390/ijms23073844 (PMC8998676; doi:10.3390/ijms23073844)
Supplement: Supplementary file 1 [file ijms-23-03844-s001.zip › Mitragynin-supplementary_final.pdf]

International Journal of *Molecular Sciences*

MDPI

Supplementary Material

Cyclodextrin charge modulates the complex formation with mitragynine: an NMR and chiroptical study

Bianka Várnai[1], Ferenc Zsila[2], Zoltán Szakács[3], Zsófia Garádi[1], Milo Malanga[4], Szabolcs Béni[1,*]

[1] Semmelweis University, Department of Pharmacognosy, Üllői út. 26, H-1085 Budapest, Hungary; bnkvarnai@gmail.com (B.V.) garadi.zsofia@pharma.semmelweis-univ.hu (Z.G.) beni.szabolcs@pharma.semmelweis-univ.hu (S.B)

[2] Institute of Materials and Environmental Chemistry, Research Centre for Natural Sciences, Budapest, Hungary; zsila.ferenc@ttk.hu (F.Z.)

[3] Gedeon Richter Plc., Spectroscopic Research Department, H-1475 Budapest, P.O.B. 27, Hungary; z.szakacs@richter.hu (Z.S.)

[4] CycloLab (last affiliation), Cyclodextrin R&D Ltd, Budapest, H-1097 Illatos út 7, Hungary; malanga@cyclolab.com (M.M.)

* Correspondence: beniszabi@gmail.com or beni.szabolcs@pharma.semmelweis-univ.hu Tel.: +36 1 317 2979

Supplementary Materials

Table S1. Complete ^{1}H and ^{13}C NMR resonance assignment for mitragynine in ppm (D_2O at pH 4.5, 600 MHz).

No.	^{1}H	^{13}C
1	6.61 (d, *J* = 7.8 Hz, 1H)	100.5
2	7.11 (d, *J* = 7.8 Hz, 1H)	123.6
3	7.01 (d, *J* = 8.2 Hz, 1H)	105.2
4	-	137.9
5	-	115.7
6	-	153.6
7	-	127.0
8	-	104.7
9<br>9′	3.16 (m, 1H)<br>3.30 (m, 1H)	20.2
10<br>10′	3.32 (m, 1H)<br>3.66 (m, 1H)	53.4
11	4.11 (d, *J* = 7.8 Hz, 1H)	62.6
12<br>12′	2.25 (m, 1H)<br>2.59 (q, *J* = 10.9 Hz, 1H)	26.6
13	3.16 (m, 1H)	36.7
14	1.96 (m, 1H)	38.2
15<br>15′	3.22 (m, 1H)<br>3.59 (d, *J* = 13.0 Hz, 1H)	55.7
16<br>16′	1.40 (m, 2H)	17.8
17	0.80 (t, *J* = 7.4 Hz, 3H).	11.2
18	-	107.7
19	-	171.0
20	3.63 (s, 3H)	51.7
21	7.58 (s, 1H)	163.4
22	3.77 (s, 3H)	61.8
23	3.84 (s, 3H)	55.3

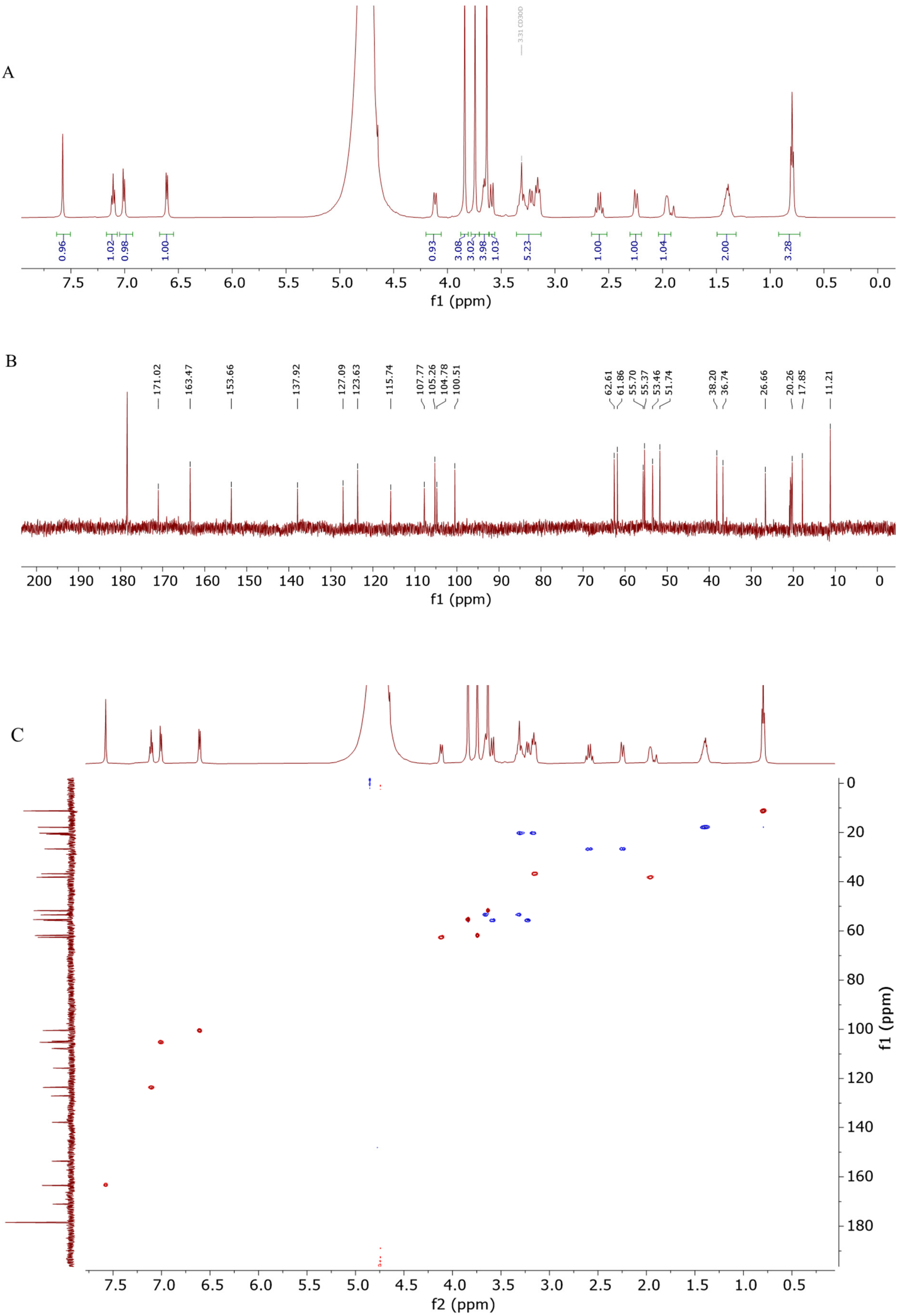

Figure S1. ^{1}H (**A**), ^{13}C (**B**) and HSQC (**C**) NMR spectra of mitragynine.

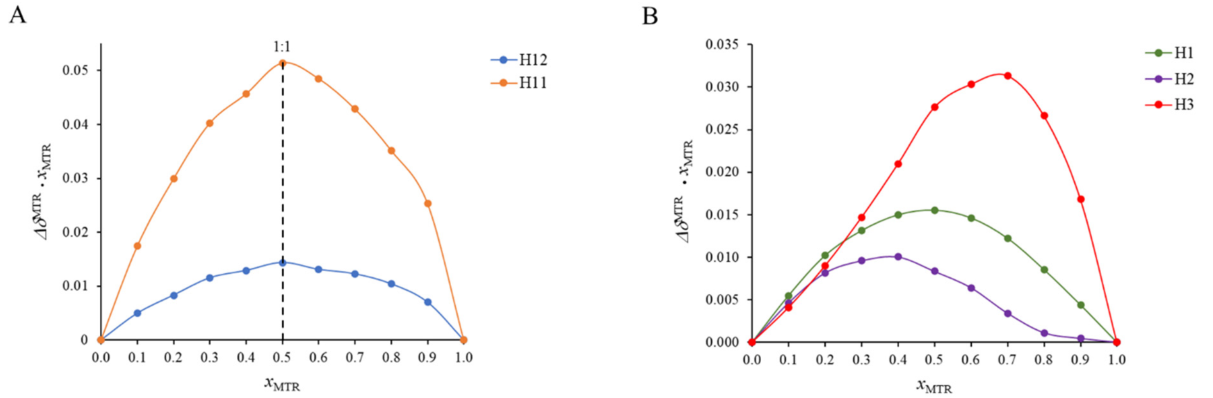

Figure S2. Job's plot for the selected ¹H resonances of MTR in the case of NMR titration with βCyD (**A**) and with SBEβCyD (**B**)

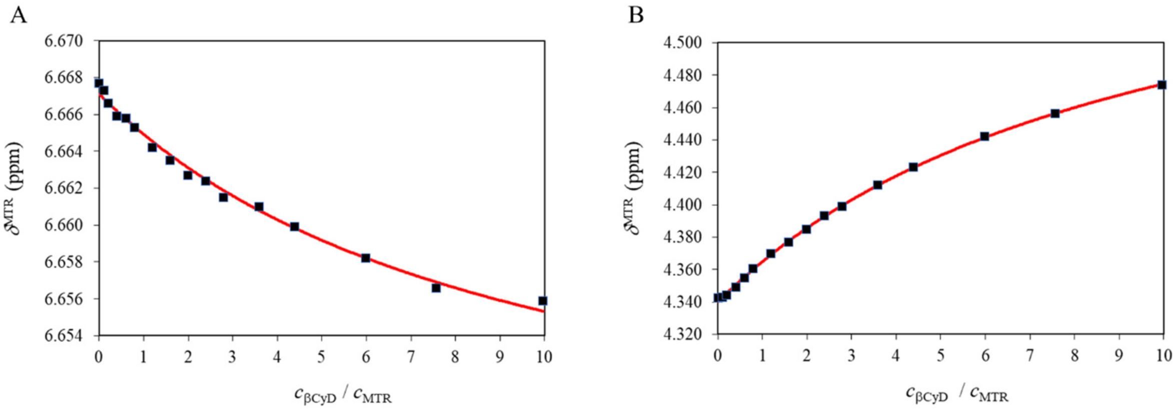

Figure S3. Chemical shift profile of the H1 (**A**) and H10 (**B**) resonances of MTR upon titration with βCyD, fitted by the 1:1 complexation model using OPIUM program (red curves).

Table S2. ¹H NMR chemical shifts (in ppm) of the uncomplexed and complexes species along with the stability constants of the 1:1 and 2:1 complexes (MTR titration with SBEβCyD) computed by simultaneous fitting of all titration profiles with the OPIUM program (uncertainties in parenthesis are estimated standard deviations for the last significant digit)

Nucleus	δ_{MTR}	$\delta_{MTR \cdot CyD}$	$\delta_{2MTR \cdot CyD}$
H1	6.6678 (12)	6.6203 (8)	6.5319 (103)
H2	7.1695 (12)	7.2312 (9)	7.1325 (56)
H3	7.0744 (12)	7.1423 (8)	7.0795 (35)
H11	4.3549 (13)	4.6067 (17)	4.0321 (328)
H12	2.3595 (12)	2.4563 (9)	2.3005 (80)
H17	0.8663 (12)	0.9225 (8)	0.8432 (46)
